# Origination of New Immunological Functions in the Costimulatory Molecule B7-H3: The Role of Exon Duplication in Evolution of the Immune System

**DOI:** 10.1371/journal.pone.0024751

**Published:** 2011-09-13

**Authors:** Jing Sun, Fengqing Fu, Wenchao Gu, Ruhong Yan, Guangbo Zhang, Zhiyong Shen, Yinghui Zhou, Han Wang, Bairong Shen, Xueguang Zhang

**Affiliations:** 1 Institute of Medical Biotechnology, Soochow University, Suzhou, China; 2 Suzhou Health Technology College, Suzhou, China; 3 Stem Cell Research Laboratory of Jiangsu Province, Suzhou, China; 4 Clinical Immunology Laboratory, First Affiliated Hospital, Suzhou, China; 5 Laboratory of Developmental Genetics and Genomics Medical College, Soochow University, Suzhou, China; 6 Center for Systems Biology, Soochow University, Suzhou, China; California State University Fullerton, United States of America

## Abstract

B7-H3, a recently identified B7 family member, has different isoforms in human and mouse. Mouse B7-H3 gene has only one isoform (2IgB7-H3) with two Ig-like domains, whereas human B7-H3 has two isoforms (2IgB7-H3 and 4IgB7-H3). In this study a systematic genomic survey across various species from teleost fishes to mammals revealed that 4IgB7-H3 isoform also appeared in pigs, guinea pigs, cows, dogs, African elephants, pandas, megabats and higher primate animals, which resulted from tandem exon duplication. Further sequence analysis indicated that this duplication generated a new conserved region in the first IgC domain, which might disable 4IgB7-H3 from releasing soluble form, while 2IgB7-H3 presented both membrane and soluble forms. Through three-dimensional (3D) structure modeling and fusion-protein binding assays, we discovered that the duplicated isoform had a different structure and might bind to another potential receptor on activated T cells. In T cell proliferation assay, human 2IgB7-H3 (h2IgB7-H3) and mouse B7-H3 (mB7-H3) both increased T cell proliferation and IL-2, IFN-γ production, whereas human 4IgB7-H3 (h4IgB7-H3) reduced cytokine production and T cell proliferation compared to control. Furthermore, both h2IgB7-H3 and mB7-H3 upregulated the function of lipopolysacharide (LPS)-activated monocyte *in vitro*. Taken together, our data implied that during the evolution of vertebrates, B7-H3 exon duplication contributed to the generation of a new 4IgB7-H3 isoform in many mammalian species, which have carried out distinct functions in the immune responses.

## Introduction

For fine-tuning immue response, several costimulatory molecules are needed including the well-known CD28 and B7 family as B7.1 B7.2 [1]. More recently, new members of B7 family have been discovered, such as B7-H3, which was cloned from a dendritic cell (DC) cDNA library [2]. Genomic analysis revealed that human B7-H3 had two isoforms, whereas mouse B7-H3 only had one isoform. Due to exon duplication, human B7-H3 gene contains four Ig-like domains, two pairs of IgV-IgC, which can be alternatively spliced into two proteins: 4IgB7-H3 (B7-H3b) containing four Ig-like domains or 2IgB7-H3 (B7-H3) with two Ig-like domains. Mouse B7-H3 gene only expresses one protein with two Ig-like domains [3].

Several studies demonstrated B7-H3 as a positive costimulatory molecule and an IFN-γ-inducer in activated human T cells [2], [4]–[8]. However, other experiments showed that B7-H3 transfectants could down-regulate T-cell proliferation and IFN-γproduction, suggesting that it also has inhibitory effect [9]–[11]. Although there were a few literatures reporting on an inhibitory role of murine B7-H3 (mB7-H3) [10], [11], a great quantity of literatures stated that mB7-H3 acted as a positive costimulatory molecule for T cell [5]–[8], [12]–[16]. For these discrepant findings, there were two possible explanations: one is whether there are two functionally distinct B7-H3 receptors on T cells, as B7.1, B7.2 could bind to T-cell receptors CD28 or CTLA-4, and thus play different functions; the other is that two isoforms of B7-H3 might express in human exerting different effects: one generating positive costimulatory signal as mB7-H3, while the other acting as an inhibitory molecule in immune responses [17]–[18].

Soluble costimulatory molecules, such as sCD28 or sPD-L1, have an important role in the costimulatory regulatory network [19]. Previously, we reported the existence of a new soluble B7-H3 (sB7-H3), which could be released from dendritic cells, monocytes and other tumor cells by matrix metalloproteinases (MMP) cleavage [20]. Our previous articles reported that the expression of circulating or regional sB7-H3 was significantly elevated in tumor or inflamed patients and was considered as a promising biomarker to help improving tumor diagnosis and infection diseases [21]–[23]. Costimulatory molecules could be generated from proteolytic cleavage, such as ICOS and PD-L1 [24], and/or generated from mRNA splicing as PD-1 and CTLA-4 [25]–[26].However, whether the sB7-H3 could be resulted from alternative mRNA splicing or whether both two isforms could produce sB7-H3 remains unknown.

In this paper, we performed a phylogenetic study of B7-H3 gene and determined the expression patterns of two known isoforms in various species. Analyzing the sequences of 4IgB7-H3, we defined a new conserved region, which is critical in determining whether soluble form of B7-H3 will be generated. Moreover, structure modeling study and fusion-binding assay suggested that 2IgB7-H3 and 4IgB7-H3 had divergent functions through binding to distinct receptors on immune cells. Our *in vitro* experiment provided the evidence that two isoforms had different functions: h2IgB7-H3 and mB7-H3 act as a positive costimulatory signal for T cells and monocytes, whereas h4IgB7-H3 serves as an inhibitory costimulator to T cells.

## Results

### Phylogenetic study of 4IgB7-H3 gene

**Figure 1:**
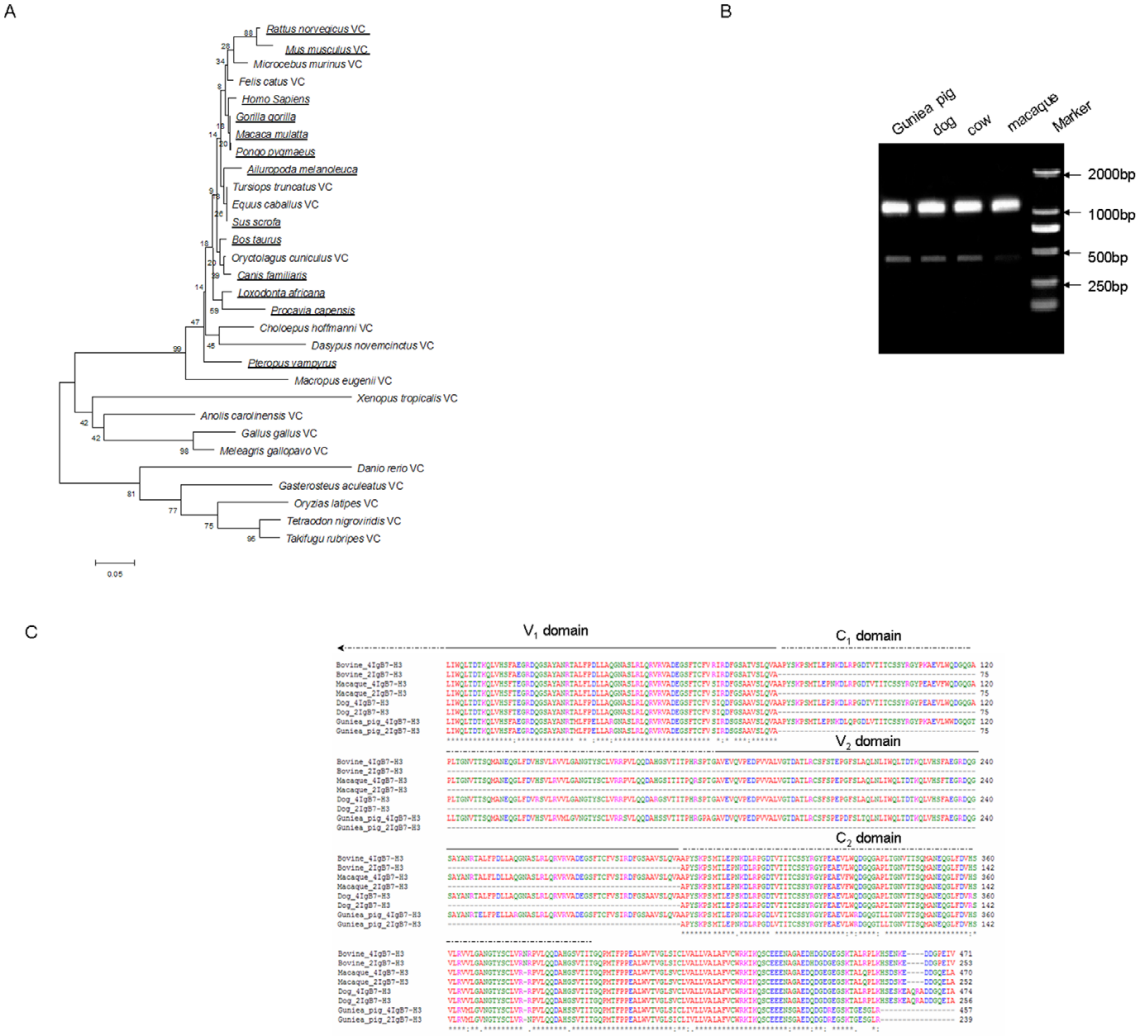
Identification and analysis of B7-H3 isoforms in different species. (A)phylogenetic analysis of the B7-H3 gene in vertebrates. The tree was constructed from CLUSTAL generated amino acid alignments using the neighbor-joining method. Tree topography was evaluated by bootstrapping 500 times with percentages shown at nodes. The species with duplication event were underlined and the species with only 2IgB7-H3 were labeling with VC after the name. (B) PCR analysis of different RNA samples using B7-H3-specific primer. A product of about 1200 bp corresponds to a 4IgB7-H3 molecule, whereas a 500 bp would represent the 2IgB7-H3 gene. (C) Sequence alignment of deduced translated cow, guinea pig, macaque and dog B7-H3 products. Dark bars or dotted lines above sequence alignment denote exon domains demarcated by genomic sequences.

In this study, B7-H3 sequences from 38 vertebrates from teleost to tetrapod species were readily retrieved using TBLASTN and the B7-H3 forms were deduced from analyzing the sequence data. It suggested that besides human, 4IgB7-H3 isoform also existed in guinea pig, cow, pig, dog, African elephant, panda, megabat, Rhesus macaque, and Chimpanzee sequence. Genes' location, exon numbers and protein length of these 4IgB7-H3 molecules are shown in Table 1. Thirty species with complete B7-H3 sequences were used to generate phylogenetic trees and the species happened duplication were underlined (Fig. 1A). In this tree, we observed that short form B7-H3 with two domains in extro-celluar region exists in pisces, ammphibia and aves which were labeled with VC after the name, while some mammalian animals have long isoform with four Ig-like domains. Although some mammalian species, as mouse and rat, have short form B7-H3 due to exons losing, two other domains' encoding sequences were found in the introns. Other mammalian animals with short form might have 4IgB7-H3 form or above-mentioned intron sequences as a result of insufficient sequences presented in public databases.

**Table 1:** Properties of 4IgB7-H3 in some vertebrates.

Species name	genome positions	Protein length and exon numbers	reference
Guinea pigs (*Cavia porcellus*)	GeneScaffold_6773: 25,646–34,702	521/7	ENSCPOT00000007869
Cow (*Bos taurus*)	Chr.10: 19,539,333–19,552,113	556/9	ENSBTAT00000026300
Dog (*Canis familiaris*)	Chr.30:39,973,976–39,987,154	538/9	GI:487638
African elephant (*Loxodonta africana*)	GeneScaffold_1416: 5,710–17,518	527/10	ENSLAFT00000014929
Rhesuls macaque (*Macaca mulatta*)	Chr.3: 27,037,697–27,032,447	534/9	ENSMMUT00000007668
Chimpanzee (*Pan troglodytes*)	Chr.15:71,496,674–71,510,990	534/9	ENSPTRT00000013400
Human (*Homo sapiens*)	Chr. 15: 73,976,554–74,006,859	534/9	ENST00000318443
marmoset *(Callithrix jacchus)*	Chr. 10: 15,898,866–16,036,078	531/10	ENSCJAT00000060454
Gorila (*Gorilla gorilla*)	Chr. 15: 53,382,251–53,415,302	534/10	ENSGGOT00000013982
Orangutan(*Pongo pygmaeus*)	Chr. 15: 71,102,366–71,116,123	534/9	ENSPPYT00000007820
Pig (*Sus scrofa*)	Chr. 7: 65,153,991–65,183,019	535/10	ENSSSCT00000002146
Panda(*Ailuropoda melanoleuca*)	Scaffold_GL192695.1:9,955–20,517	531/8	ENSAMEG00000005720
Megabat(*Pteropus vampyrus*)	GeneScaffold_718:10943–24975:1	529/9	ENSPVAP00000014464

The accession number with first letter “E” are Ensembl ID from Ensembl database and GI is from GenBank database.

We performed RT-PCR to amplify B7-H3 from the representative species (Fig. 1B). Two unambiguous bands were detected in the guinea pig, dog, cow and macaque, with one predominant band around 1200 bp, representing the predicted size of the 4IgB7-H3 and a minor band of 500 bp corresponded to the predicted size of 2IgB7-H3. All bands were sequenced and confirmed to be B7-H3 genes. We performed sequences analysis between 4IgB7-H3 and 2IgB7-H3 and found that two forms of B7-H3 transcripts were produced by alternative splicing from the B7-H3 gene (Fig. 1C).

### Sequence analysis of this new 4IgB7-H3 isoform

**Figure 2:**
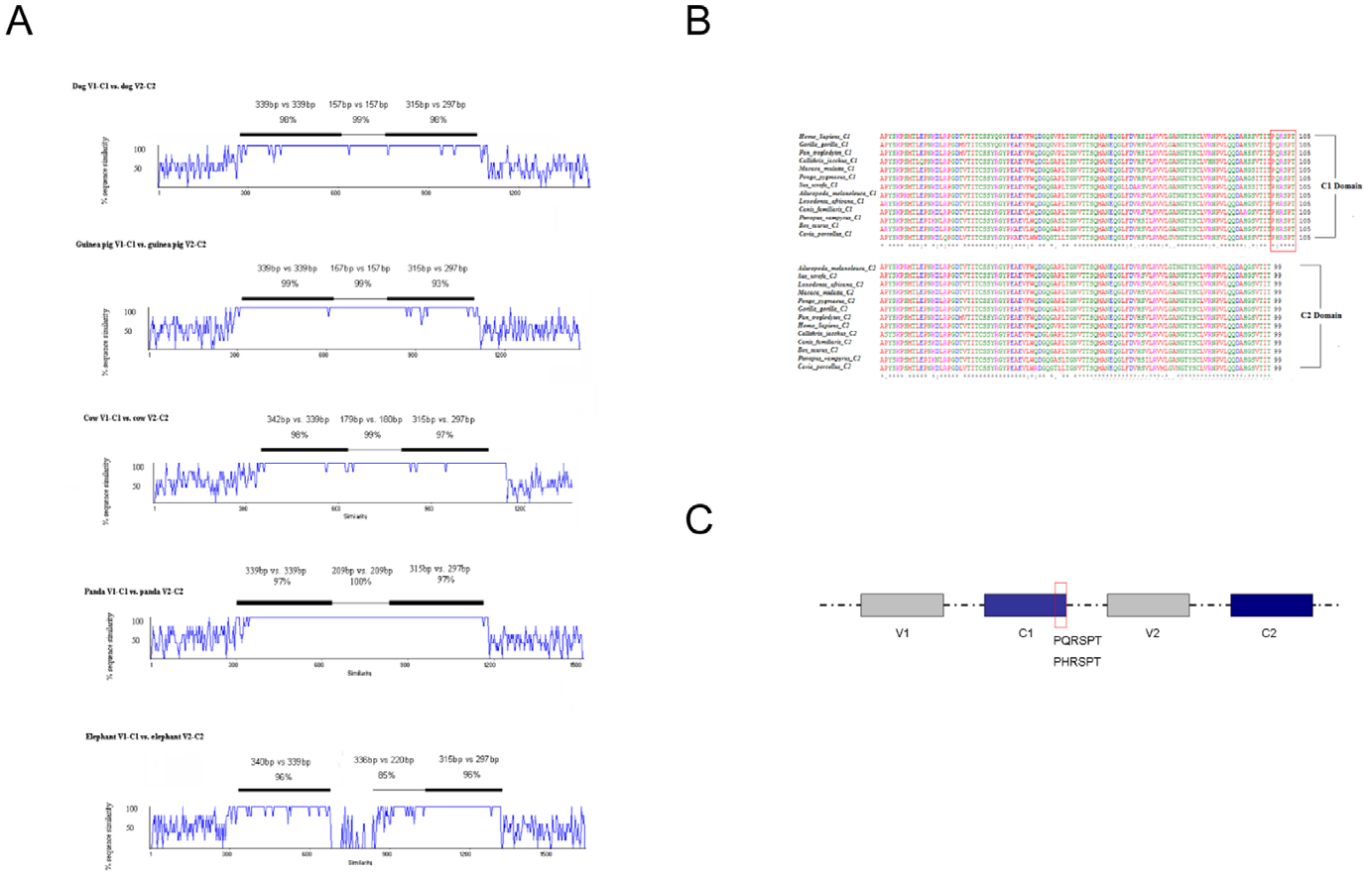
Sequences analysis of 4IgB7-H3. (A) Interspecies alignment histograms of VC duplications of guinea pig, cow, dog, elephant, and panda animals' loci. Sizes of exon domains and percentage sequence identities are as indicated. (B) Multiple sequence alignments of the first (C_1_) and second (C_2_) IgC domains from the vertebrates having 4IgB7-H3 isoform. The accession numbers are shown in Table 1.The c-like domains' position are acquired form ensemble databases. The amino acids in red line are special and redundancy in the first C domain. (C) The location of the special region in 4IgB7-H3.

Human B7-H3 locus on chromosome 16 consisted of four Ig-like domains: V_1_, C_1_, V_2_, and C_2_. V_1_C_1_ share 96% sequence homology with V_2_C_2_. Based on its genomic sequence data, B7-H3 underwent exon duplication leading to tandem repeated VC domains (4IgB7-H3). We also observed the same event in other species. Here, we analyzed 4IgB7-H3 DNA sequences in the dog, cow, elephant, panda and guinea pig. The comparison between two pairs of VC exons and introns were shown in Figure 2A. Ling *et al*. had analyzed the interspecies alignment in human [3], and we got similar results in human and other primate animals (data not shown). Moreover, we found a conserved region (PQRSPT or PHRSPT) in the C terminus of the first IgC domain (Fig. 2B), which was absence in 2IgB7-H3. It might be produced by splicing donor site in the exon duplication event.

### Soluble B7-H3 shedding from 2IgB7-H3

**Figure 3:**
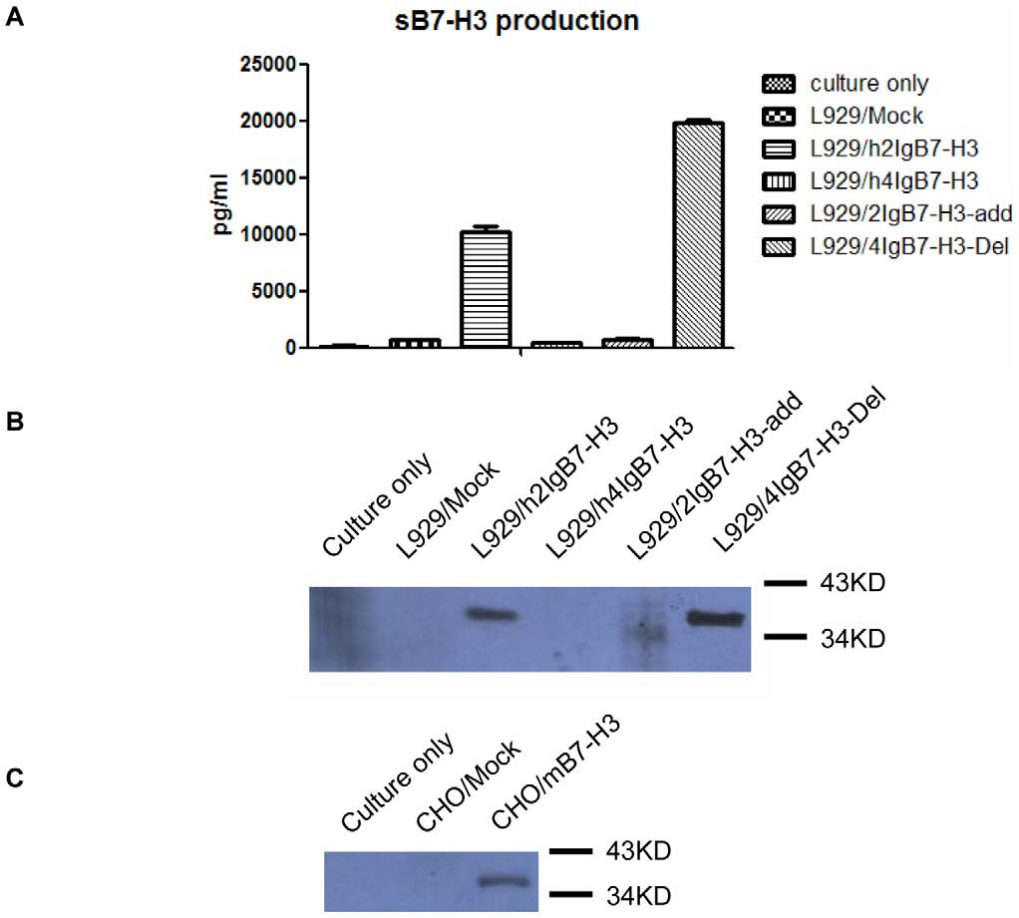
Soluble B7-H3 was released from h2IgB7-H3, h4IgB7-H3-Del and mB7-H3, not from h4IgB7-H3 and h2IgB7-H3-Add genes. (A) ELISA detecting of the culture supernatant of transfected cell lines (B) sB7-H3 of human releasing in transfected cells supernatant was detected by western blot analysis (C) mouse sB7-H3 was determined in transfected supernatant cells by western blot analysis.

As previously described, we developed an ELISA kit for the measurement of sB7-H3 [21], which could be used for sB7-H3 measurement in serum and tumor cell culture supernatants. We observed that sB7-H3 was released from 2IgB7-H3-transfected L929 cell line, but not in 4IgB7-H3-transfected cell line. As shown in Fig. 3A, an average level of sB7-H3 in L929/h2IgB7-H3 supernatants was 10.18 ng/ml, while no difference was detected between L929/h4IgB7-H3 and L929/mock supernatants. Western blot analysis was performed to determine the molecular weight of sB7-H3 and a band near 37 kDa was detected in human 2IgB7-H3- and mouse B7-H3-transfected cell supernatants (Fig. 3B and Fig. 3C), as well as in lung cancer, uterine cervix cancer and colon cancer cell line supernatants (data not shown). In our previous paper [20], we reported the size of soluble B7-H3 form was around 16.5 kDa using biotinylated mAb 21D4 (prepared by our lab). In this studies, we revised the size as approx. 37kDa using various antibodies purchased from companies or prepared by our lab.

Our previous reports have verified that sB7-H3 could be released from cell membrane protein by MMP digestion. Judged from the size of the cleavage product of sB7-H3, the cleavage site should be close to the trans-membrane region. Both B7-H3 isoforms had the same sequence in this region, but the soluble form could only be cleaved from 2IgB7-H3. Compared with 2IgB7-H3, 4IgB7-H3 has two pairs of V, C domains and the sequence similarity between pairs exceeds 98% [3] except a region (PQRSPT or PHRSPT) in the C terminus of the first IgC (Fig. 2B). So we presumed that the new region might lead to B7-H3 remodeling, which block the cleavage effect of MMP enzyme. To verify the effect of conserved PQPSPT sequence, PQPSPT-deleted 4IgB7-H3 (4IgB7-H3-Del) and PQRSPT-added 2IgB7-H3 (2IgB7-H3-Add) plasmids were constructed and transfected to L929 cell line. Soluble B7-H3 was identified in the 4IgB7-H3-Del culture supernatants by ELISA and Western blot, while it was absent in 2IgB7-H3-Add culture supernatants (Fig. 3). In addition, more sB7-H3 was detected from 4IgB7-H3-Del compared to 2IgB7-H3.

### Different 3D structures of two B7-H3 isforms

**Figure 4:**
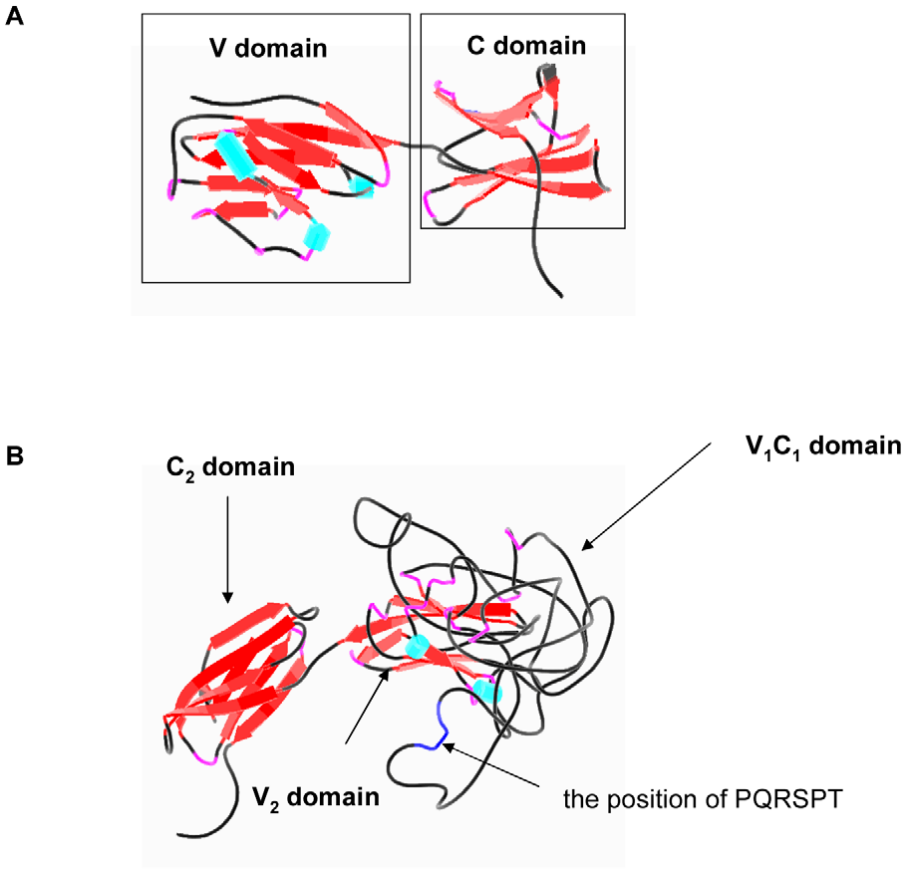
Three-dimensional (3D) Structural models for B7-H3 proteins were proposed by homology modeling using the crystal structure of known PD-L1. (A) the model of 2IgB7-H3 (B) the model of 4IgB7-H3.

Exon duplication and alternative splicing of B7-H3 locus resulted in two similar splicing products, 4IgB7-H3 and 2IgB7-H3, which shares over 95% sequence homology. Some researchers have postulated that these two similar B7-H3 isoforms bind to distinct receptors with opposing functions. The 3D structures of these two proteins may shed light on the possible binding receptors. Through PSI-Blast, we acquired PD-L1 x-ray structure (3biSA)[27], which was close to B7-H3 amino acid sequence (34% identity) and applied to our modeling (Fig. 4). We observed that 4IgB7-H3 has different numbers of helices and beta, and different conformation compared with the 2IgB7-H3 isoform, and the tandem duplicated domain V_1_C_1_ and V_2_C_2_ did not show similar 3D structure. In the 4IgB7-H3 model we could find the above mentioned PQRSPT sequence was located in one loop region, which might have an important role in remaining the structure.

### Two putative receptors for B7-H3 on the surface of activated T cells

**Figure 5:**
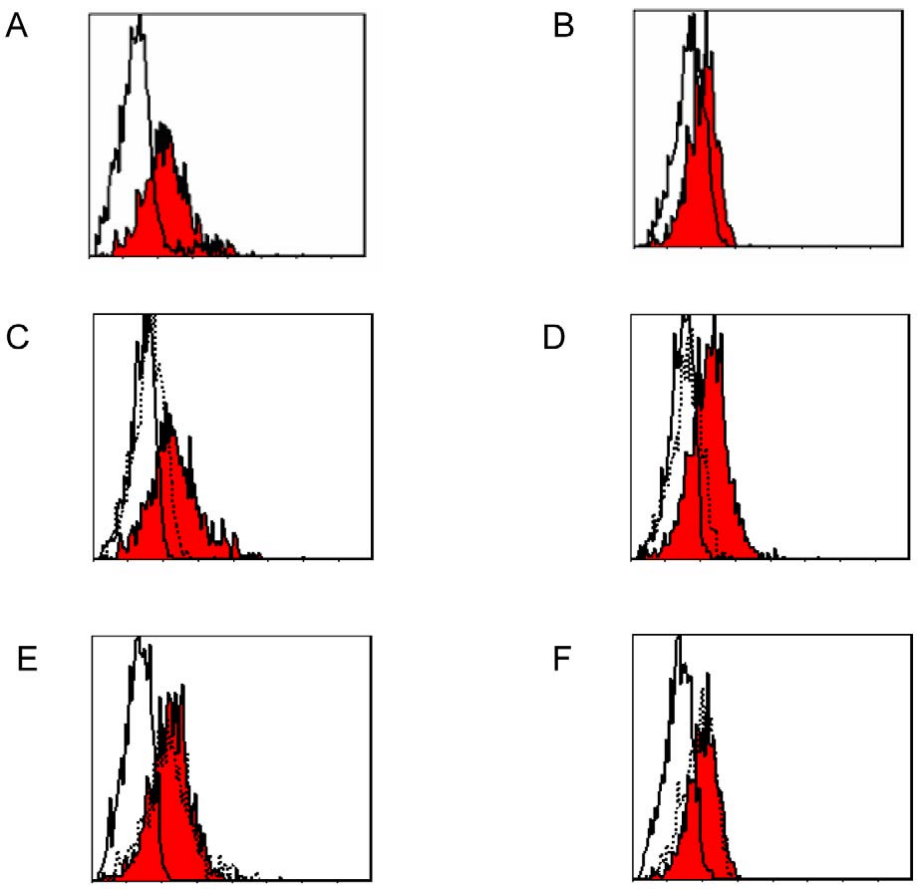
Surface expression of a putative receptor for B7-H3 on human activated T cells. Human PBMC from healthy donors (n = 5) were incubated with PHA (30 µg/ml) for up to 24 h. Data shown are one representative of five independent experiments. (A)(B) Cells were stained with biotinylated h2IgB7-H3Ig or h4IgB7-H3Ig (1 µg) (red filled) or biotinylated human IgG (1 µg) as the control (grey), followed by dual-staining with PE-conjugated streptavidin and FITC-conjugated anti-CD3 mAb. (C)(D) Data are presented with 10 µg non-biotinylated 2/4IgB7-H3-Ig followed by 1 µg biotinylated 2/4IgB7-H3-Ig (grey dash line) or biotinylated 2/4IgB7-H3-Ig (red filled) or biotinylated human IgG (100 ng) staining as the control (grey). (E)(F) Data are presented with 10 µg non-biotinylated 4/2IgB7-H3-Ig followed by 1 µg biotinylated 2/4IgB7-H3-Ig (grey dash line) or biotinylated 2/4IgB7-H3-Ig (red filled) or biotinylated human IgG (100 ng) staining as the control (grey).

Although a definite counter-receptor for B7-H3 has not been clarified, most published reports suggested that B7-H3 binds to a putative receptor expressed on PHA- or anti-CD3 mAb-activated T cells [2], [3]. To assess whether two isoforms of B7-H3 binds to different receptors on activated T cells, cells were pre-incubated with non-biotinylated proteins, and biotinylated h2IgB7-H3Ig/ h4IgB7-H3Ig were applied to examine a putative B7-H3 receptor. We found both biotinylated proteins bound to isolated CD3^+^ cells from PHA-activated PBMC (Fig. 5A and Fig. 5B). Pre-incubation with the same protein without biotin-labeling significantly inhibited the binding (Fig. 5C and Fig. 5D), while incubation with another excess non-biotinylated protein had no effect on the binding (Fig. 5E and Fig. 5F). Taken together, these results suggested two receptors might exist in activated T cells.

### The effect of B7-H3 on T cells activation

**Figure 6:**
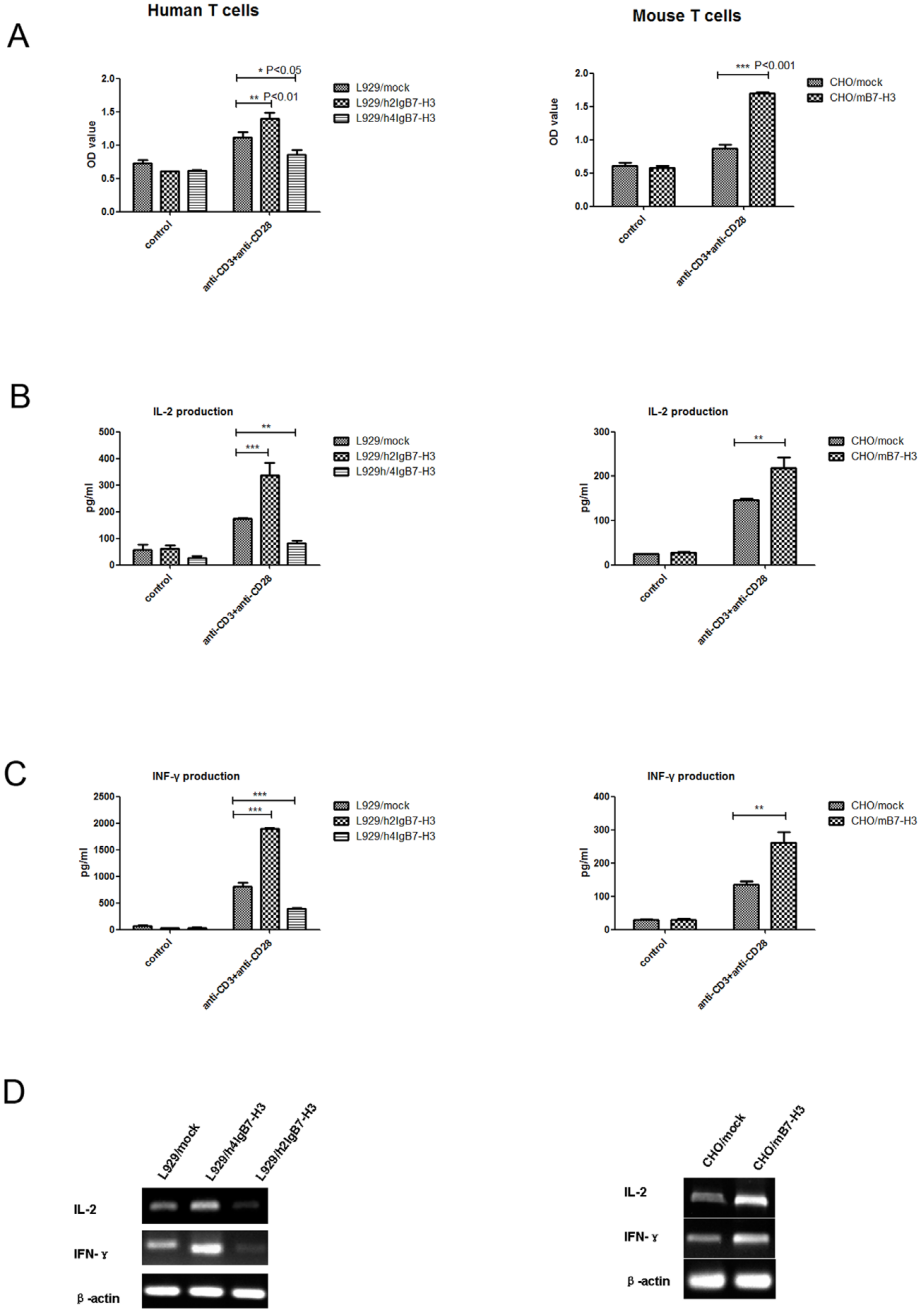
Costimulation of T cell response by h2IgB7-H3 and mB7-H3 while inhibition of T-cell activation by h4IgB7-H3. (A) Human T cells and mouse T cells were co-cultured with L929 transfectants or CHO transfectants (ratio 10 1) stimulated with plate-bound anti-CD3 mAb and soluble anti-CD28 for 72h. T cell counting was analyzed by cck-8. The data are representative for six independent experiments (B), (C) Culture supernatant was harvested after 72h and subjected to IL-2, IFN-γ measurement.(D) Expression of the IL-2 and IFN-γ mRNA level in human and mouse T cells with different transfected cells. β-acin was used as the control.

L929/h2IgB7-H3 and CHO/mB7-H3 treatment significantly enhanced the proliferation of T cells activated by anti-CD3 and anti-CD28 Ab (Fig. 6A), and increased both mRNA and protein expression of IL-2 and IFN-γ (Fig. 6B and Fig. 6C). In contrast, 4IgB7-H3 treated T cells had decreased proliferative responses (Fig. 6). Anti-B7-H3 Ab (prepared by our lab) at 1 µg/ml could partially rescue the B7-H3 costimulatory or inhibitory effect on proliferation (data not shown).

### The effect of h2IgB7-H3 and mB7-H3 on LPS-activated monocytes

**Figure 7:**
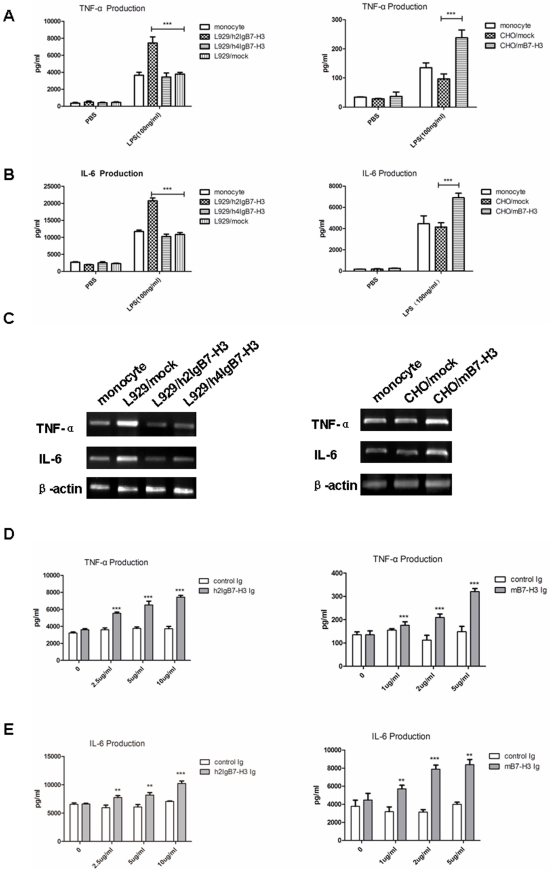
Human 2IgB7-H3 and mouse B7-H3 argument LPS-induced proinflammatory cytokine release. (A) and (B) is the TNF-α or IL-6 production of human monocytes or mouse monocytes cocultured with various transfectants. (C) Expression of the TNF-α or IL-6 mRNA level in human and mouse monocytes with different transfected cells. β-acin was used as the control. (D) and (E) TNF-α or IL-6 were up-released with the indicated dose of h2IgB7-H3 or mB7-H3 protein.

Previous, we reported that sB7-H3 was elevated in patients with bacterial infections and its level was correlated with plasma TNF-α, which suggested that B7-H3 might be involved in inflammatory reaction [18]. Interestingly, we detected a putative receptor for B7-H3 on monocytes and peritoneal macrophages from septic patients in the previous study. Here, we stimulated human peripheral monocytes with various doses of 2IgB7-H3, 4IgB7-H3-Ig-fusion protein or various numbers of 2IgB7-H3, 4IgB7-H3/L929 cells in the presence of LPS. We also stimulated murine monocytes from spleen with mB7-H3 protein or mB7-H3/CHO cell. Human 2IgB7-H3 and murine B7-H3, significantly augmented the mRNA expression and the release of LPS-stimulated TNF-α, IL-6 (*p*<0.05) in a dose-dependent manner (Fig. 7), while human 4IgB7-H3 had no effect.

## Discussion

The evolution of immunological genetics and functions is critical to understand current immunological biology in human and related mammals. Here we examined the evolution of a costimulatory molecule B7-H3, which is a new member of the B7 family, and has two isoforms in humans while only one isoform in mice [3], [17]–[18]. Through interrogating various genomic databases, we observed that B7-H3 genes in pisces, ammphibia and aves had a short form with single V and C domains(Fig 1A), while the long form 4IgB7-H3 with pairs of VC domains was presented in many mammals for example guinea pigs, dogs, cows, pigs, elephants, pandas, megabats and higher primates(Table 1, Fig 1B). Further genomic data analysis in these species revealed that the B7-H3 locus underwent exon duplication leading to the emergence of 4IgB7-H3 with tandem repeated VC domains (Fig 2A). In evolution, the segment of B7-H3 gene including two exons coding VC domain and an intron happened cross over and then recombination occurred. Judged from the B7-H3 phylogenetic tree and genomic sequence data we presumed this DNA segment duplication event might happen from the time prior to the emergence of mammalian animals∼300 million years ago[29]. This time couldn't be evident before all mammalian species' B7-H3 sequencing was completed. Although duplication event also happened in some mammalian animals i.e., mouse and rat, they had only the single VC form due to exon losing and the sequence encoding the other putative C_1_ and V_2_ exons were presented in the intron region. Based on the comparison between V_1_C_2_ and its duplication product C_1_V_2_, we found some nonsynonymous mutation (data not shown) and a new region in the C_1_ domain, which suggested that the tandemly duplicated exons were not redundant and might have new biology functions It was important to discuss about the B7-H3 exon losing happened in rodent lineage and we proposed that it might be caused by the absence of the corresponding receptor of 4IgB7-H3 in these animals, which could be further investigated after the B7-H3 potential receptor is identified. The expression of 4IgB7-H3 in several mammalian animals was confirmed by RT-PCR. We revealed that 2IgB7-H3 transcript could be produced by alternative splicing of B7-H3 gene in these animals and the predominant transcript was 4IgB7-H3, which was consistent with studies in human [3], [17]–[18], [30]. These results indicated exon duplication in B7-H3 evolution created a new isoform and this study is designed to investigate the function of the new isoform.

Analyzed the 4IgB7-H3 sequences, there is a conserved region in the C terminus of first IgC domain of various vertebrates' 4IgB7-H3, i.e., PQRSPT or PHRSPT, which was present in 4IgB7-H3 and absent from 2IgB7-H3. This region determines the presenting form and functions of 4IgB7-H3, which is observed in the following data. Our previous work determined that sB7-H3 was released from membrane protein mediated by MMP digestion; and in the present study, we determined that the molecular weight of sB7-H3 is around 37KD by Western blotting, which was much close to the size of 2IgB7-H3 protein. Since there was no soluble form in the 4IgB7-H3-transfected supernatant, we proposed that 2IgB7-H3 was the only form to produce sB7-H3. Furthermore, we generated a 4Ig-like DNA construct without this conserved region or a 2Ig-like DNA construct with this region. Interestingly, sB7-H3 could not be cleaved from membrane protein if PQRSPT amino acids were added and soluble form could only be produced when these amino acids were removed. These results indicated that the present of this region might interfere in the MMP cleavage. Judged from the size of sB7-H3, we presumed the cleavage site should be close to the 2IgB7-H3 trans-membrane region. As two isoforms of B7-H3 had the same sequences in the region adjacent to transmembrane region, we speculated that PQRSPT motif might change the protein structure to protect the site from MMP cleavage. In addition, proline was often very flexible and having special role in the structure of protein, which might give a support to the B7-H3 deformation [31]. When this region was removed from 4IgB7-H3, two MMP cleave sites was exposed, so we could found greater amount of soluble form. The soluble form cleaved from 2IgB7-H3 and 4IgB7-H3-Del had the similar molecule weight.

B7-H3 was originally found be able to enhance T cell proliferation, cytotoxicity and IFN-γproduction [2], [4]. However, other studies indicated that B7-H3 inhibited the activation of T cells and cytokine production [9], [11]. Moreover, a recent study showed that the effects of B7-H3 on T cells depended on the activation state of the T cells and B7-H3 had different effect on TCR-activated T cells, resting T cells and cytokine-activated T cells [28]. Studies with different results might suggest that two isoforms of B7-H3 have evolved divergent functions in immune responses through binding to different receptors. So we examined the costimulatory activity of human and mouse B7-H3 *in vitro*. Interestingly, we found that h2IgB7-H3 and mB7-H3 enhanced T cell proliferation and increased IFN-γ and IL-2 releasing, while h4IgB7-H3 down-regulated the activation of T cells (Fig. 7). Differential 3D structure models of two isoforms and fusion-protein binding assays on activated T cells also support the notion of the potential presence of two receptors for B7-H3 with opposing functions. To our knowledge, this is the first report providing both experimental and bioinformatic evidence to support the existence of two receptors for B7-H3 ligand.

Our previous study showed that significantly elevated levels of sB7-H3 in the circulation were observed in patients diagnosed with sepsis or bacterial meningitis [22]. Furthermore, levels of sB7-H3 in septic patients were correlated with their clinical outcomes and circulating proinflammatory cytokines TNF-α and IL-6. In addition, a putative receptor for B7-H3 was detected on monocytes and peritoneal macrophages from septic patients. These reports indicated that costimulatory molecule B7-H3 might play an important role in infection immune. In this study we demonstrated that h2IgB7-H3 and mB7-H3 are able to increase the TNF-αand IL-6 production medicated LPS signaling. However, no similar effect was observed in h4IgB7-H3. These results argue that the functions of 2IgB7-H3 protecting against harmful infection require the ubiquitous existence of 2IgB7-H3 in lower vertebrates to primate animals.

In summary, we performed in this work, for the first time, a systemic analysis of the 2IgB7-H3 and 4IgB7-H3 expression in various vertebrates and found duplication event happened in most lineages of vertebrates. 2IgB7-H3 existing in all species could take part in the infection immune in membrane-bound or soluble form and upregulate the function of T cells and monocytes. In contrast, 4IgB7-H3 could only inhibit T cell response through binding a different receptor, which needs to be verified through further experiments after the receptors were found.

## Materials and Methods

### The Ethics Statement

Prior to commencing these experiments in this study, the approval from the Ethics Review Board of Soochow University was granted and the written informed consent was obtained from each blood donor. In addition, the animal experiments in this study were carried out in strict accordance with the recommendations in the Guide for the Care and Use of Laboratory Animals of the National Institutes of Health and the protocol was approved by the Ethics Review Board of Soochow University. (Ethics permit number of this study is 200895). All surgery in animal experiments was performed under sodium pentobarbital anesthesia, and all efforts were made to minimize suffering.

### Identification and sequence analysis of B7-H3

B7-H3 or B7H3 or CD276 were used as queries to be searched in several genome databases including GenBank (http://blast.ncbi.nlm.nih.gov/Blast.cgi) ENSEMBL databases (http://www.ensembl.org/index.html) and the Institute for Genomic Research (http://compbio.dfci.harvard.edu/tgi/tgipage.html). ESTs representing partial sequences were used to search EST indices using BLASTn and overlapping sequences were assembled using the Assembler function of MacVector. Individual genomes were interrogated using TBLASTN at ENSEMBL (www.ensembl.org/index.html) or BLAT (http://genome.ucsc.edu/cgi-bin/hgBlat). Phylogenetic trees were generated using MEGA4 software (www.megasoftware.net; Kumar et al., Arizona State University, Tempe, AZ, USA). The exon and intron sequences of selected vertebrates were aligned using Align-X ClustalW module of Vector NTI version 7.1 (Informax Inc., North Bethesda, MD, USA). Multiple amino acid sequence alignments were performed using ClustalW.

### 3D structure of two isoforms of human B7-H3

The modeling of the 3D structure of these two proteins was performed by three automated homology modeling programs, Geno 3D, Swissmodel and Modeller [32]. Briefly, PSI-BLAST (http://blast.ncbi.nlm.nih.gov/Blast.cgi) used for template structure search. 3D structure of PD-1/PD-L1 complex also named 3bisA was selected as template sequence. The template was submitted to Geno3D and Swissmodel for automated homology modeling. For Modeller, the template and target sequences were carefully aligned to remove potential alignment errors. The overall stereochemical quality of the protein was assessed by Ramchandran plot analysis. The validation for structure models obtained from the three software tools was performed by PROCHECK. The structures were visualized using Swiss PdBviewer v 4.0.1.

### Detection of B7-H3 isoforms in the guinea pig, dog, cow and macaque by quantitative reverse transcription PCR

RNAs were extracted from the spleen of guinea pig, dog and cow using TRIzol reagent (Takara, Japan). And cDNAs were converted by an Oligo(dT) primer using primescript First cDNA synthesis kit. The template used for macaque's B7-H3 amplification was extracted from Phytohaemagglutinin (PHA 30 µg/ml) -activated peripheral blood mononuclear cell (PBMC). PCR primers were designed to amplify the conserved parts of the B7-H3 sequence. PCR products were subcloned into the PMDT-9 (Invitrogen, USA) vector and sequenced. Specific primers designed upon the sequence of various species were used to characterize the predominant product of B7-H3 in the guinea pig, dog,macaque and cow.

### Construction of 4IgB7-H3-Del and 2IgB7-H3-Add plasmids

Based upon the alignments of 4IgB7-H3 protein sequences, we found a conserved region in the C terminus of first IgC domain. Two pairs of primers were designed with the 3′ one phosphorylated in the first pair primer and 5′ one phosphorylated in the second pair primer to amplify the two fragments of 4IgB7-H3. Then PCR products were purified and ligated with T_4_ ligase enzyme (Takara, Japan) and named 4IgB7-H3-Del. 2IgB7-H3 gene was added with a redundant sequence encoding PQRSPT through overlap PCR and named 2IgB7-H3-Add. After been sequenced, DNAs were subcloning into pRIES2 vector to make pRIES2/4IgB7-H3-Del and pRIES2/2IgB7-H3-Add construct. The new plasmids were transfected into the murine fibrosarcoma cell line L929 with Lipofectamine 2000 (Invitrogen) and the stable cell lines were acquired.

### Cell culture, Antibody and Ig-fusion proteins

L929/h2IgB7-H3/ [33], L929/h4IgB7-H3 [30] and CHO/mB7-H3 (unpublished paper) transfected cell lines were established previously by our colleagues. Cells were cultured in 10% fetal calf serum (FCS)(Gibco, USA)/RPMI-1640 in a 5% CO_2_, 37°C incubator. Meanwhile, the murine fibrosarcoma cell line L929, Chinese hamster ovary cell line CHO (purchased from American Tissue Culture Collection) and mock transfected cells were also cultured as controls. When these cells were incubated for a phase of growth, the supernatants were collected and frozen at −80°C.

The goat-anti-human B7-H3 antibody and goat-anti-mouse B7-H3 antibody were purchased from R&D systems. The human 2IgB7-H3-Ig-fusion protein and 4IgB7-H3-Ig-fusion protein were constructed and prepared by our lab.

### Analysis of two putative receptors in activated T cells

The PBMC were isolated by Ficoll-Hypaque through gradient centrifugation from peripheral blood of healthy donors (n = 5, Suzhou Central Blood Bank, Suzhou, China) and were activated using PHA (30 µg/ml) for 24 h. Cells were stained with biotinylated h2IgB7-H3Ig (1 µg/ml) or biotinylated h4IgB7-H3Ig (1 µg/ml). The other groups were stained with biotinylated h2IgB7-H3Ig (1 µg/ml) after incubated with non-biotinylated h2IgB7-H3 (10 µg/ml) or h4IgB7-H3 (10 µg/ml). There were another groups stained with biotinylated h4IgB7-H3Ig (1 µg/ml) after incubated with non-biotinylated h4IgB7-H3 (10 µg/ml) or h2IgB7-H3 (10 µg/ml). Biotinylated human IgG (1 µg/ml) was used as the control. All groups were followed by dual-staining with PE-conjugated streptavidin and FITC-conjugated anti-CD3 mAb. Cells were resuspended in PBS and analyzed by FACScan and CellQuest software (Becton Dickinson, San Jose, CA). Prior to commencing this study, the approval from the Ethics Review Board of Soochow University was granted and the written informed consent was obtained from each blood donors.

### Soluble B7-H3 (sB7-H3) and cytokine measurement

The supernatants of L929, L929/mock, L929/h2IgB7-H3/, L929/h4IgB7-H3, L929/4IgB7-H3-Del and L929/2IgB7-H3-Add cells were collected and analyzed by sB7-H3 ELISA kit (generated by our laboratory) [21]. Concentrations of human and murine TNF-α, IL-6,IL-2 and IFN-γ in the supernatants of LPS-stimulated monocyte and activated T cells were assessed using ELISA kit (R&D systems, USA).

### Western blotting

The supernatants of L929, L929/mock, L929/h2IgB7-H3/, L929/h4IgB7-H3, L929/4IgB7-H3-Del,L929/2IgB7-H3-Add,CHO/mock and CHO/mB7-H3 cells were collected and centrifuged at 12,000×*g* for 15 min at 4°C. These supernatants were separated by SDS-PAGE, and electrotransferred onto Immobilon-P membrane (Millipore). Membrane was treated with 1 µg/ml goat anti-mouse or goat anti-human B7-H3 Ab (R&D Systems, USA), then membranes were washed three times with wash buffer before development with rabbit anti-goat-HRP conjugate (Molecular Probes) at 0.2 µg/ml for 1 h at 4°C. Membranes were developed by chemiluminescence ECL (Amersham Biosciences).

### Isolation and treatment of human and mouse T cells

The PBMC were isolated by the same method as described above. Human T cells were isolated from PBMC using a human T Cell Enrichment Kit (Stem cell, Canada). The purity was more than 98%, identified by anti-CD3 staining. L929/mock, L929/h2IgB7-H3/ and L929/h4IgB7-H3were treated with mitomycin (1 mg/ml, Sigma, USA) for 30 min, and washed three times with PBS. T cells (10^5^ cells/well) were cultured in flat-bottom 96-well plates with L929 transfectants (10^4^ cells/well) and soluble anti-CD28 antibody (5 µg/ml, prepared by our Lab). The 96-well plates were coated with anti-CD3 antibody (0.5 µg/ml, Immunotech Co., France) at 4°C overnight. Proliferation was determined using Cell Counting Kit-8 (Dojindo Japan) for the last 5 hours of a 72-h incubation period. Supernatants were harvested at 72 h and assayed by multiplex ELISA screening (R&D systems, USA) to measure cytokine production.

Mouse spleen was dissected and put into an ice-cold Hanks balanced salt solution to generate a single cell suspension. Mouse T cells were isolated using EasySep® Mouse T Cell Enrichment Kit (Stem cell, Canada). The purity was more then 90%, identified by anti-CD3 staining. Then Mock/CHO and mB7-H3/CHO were treated as described in human T cells.

To determine the mRNA levels of cytolines, total RNA of human and mouse T cells treated with L929/mock, L929/h2IgB7-H3/, L929/h4IgB7-H3, CHO/mock and CHO/mB7-H3 were extracted and subjected to RT-PCR. Reverse transcription Reaction products were amplified with the primer pairs for IL-2 and INF-γ. As the keeping gene, PCR was also performed with β-actin primers.

### Isolation and treatment of human and mouse monocytes

CD14 positive selection kit (Stem cell, Canada) was used to isolate human monocytes from PBMC and the purity of the monocyte was more than 95% as identified by anti-CD14 staining. After treated with 100 ng/ml LPS (Sigma), cell suspensions were added to flat-bottom 96-well plates at a density of 10^5^ cells/well, and plates were incubated with L929 transfectants or B7-H3-Ig-fusion protein at 37°C and 5% CO_2_ for 18 h. Supernatants were harvested and frozen at −80°C until assayed.

CD11b^+^ cells were separated from splenocyte suspensions using a mouse CD11b positive selection kit (Stem cell, Canada). The purity of the prepared monocytes was above 95% as identified by anti-CD11b staining. Cells were stimulated with LPS (100 ng/ml) and with CHO/mB7-H3 cells or mouse B7-H3-Ig-fusion protein for 18 h. Supernatants were harvested and frozen at −80°C until assayed. In addition the monocytes were collected to extract total RNA.

### Statistical analysis

All data are expressed as mean±SD. Statistical analysis was performed with the Student's *t* test and ANOVA. Correlations were evaluated by the Pearson Correlation test. Values with *p*<0.05 were considered statistically significant.

## References

[1] Coyle AJ, Gutierrez-Ramos JC (2001). the expanding B7 superfamily: increasing complexity in costimulatory signals regulating T cell function.. Nat Immunol.

[2] Chapoval AI, Ni J, Lau JS, Wilcox RA, Flies DB (2001). B7-H3: a costimulatory molecule for T cell activation and IFN-gamma production.. Nat Immunol.

[3] Ling V, Wu PW, Spaulding V, Kieleczawa J, Luxenberg D (2003). Duplication of primate and rodent B7-H3 immunoglobulin V- and C-like domains: divergent history of functional redundancy and exon loss.. Genomics.

[4] Zhang GB, Chen YJ, Shi Q, Ma HB, Ge Y (2004). Human recombinant B7-H3 expressed in E. coli enhances T lymphocyte proliferation and IL-10 secretion in vitro.. Acta Biochim Biophys Sin (Shanghai).

[5] Wang L, Fraser CC, Kikly K, Wells AD, Han R (2005). B7-H3 promotes acute and chronic allograft rejection.. Eur J Immunol.

[6] Luo L, Chapoval AI, Flies DB, Zhu G, Hirano F (2004). B7-H3 enhances tumor immunity in vivo by costimulating rapid clonal expansion of antigen-specific CD8+ cytolytic T cells.. J Immunol.

[7] Hashiguchi M, Kobori H, Ritprajak P, Kamimura Y, Kozono H (2008). Triggering receptor expressed on myeloid cell-like transcript 2 (TLT-2) is a counter-receptor for B7-H3 and enhances T cell responses.. Proc Natl Acad Sci U S A.

[8] Sun X, Vale M, Leung E, Kanwar JR, Gupta R (2003). Mouse B7-H3 induces antitumor immunity.. Gene Ther.

[9] Leitner J, Klauser C, Pickl WF, Stockl J, Majdic O (2009). B7-H3 is a potent inhibitor of human T-cell activation: No evidence for B7-H3 and TREML2 interaction.. Eur J Immunol.

[10] Suh WK, Gajewska BU, Okada H, Gronski MA, Bertram EM (2003). The B7 family member B7-H3 preferentially down-regulates T helper type 1-mediated immune responses.. Nat Immunol.

[11] Prasad DV, Nguyen T, Li Z, Yang Y, Duong J (2004). Murine B7-H3 is a negative regulator of T cells.. J Immunol.

[12] Kobori H, Hashiguchi M, Piao J, Kato M, Ritprajak P (2010). Enhancement of effector CD8+ T-cell function by tumour-associated B7-H3 and modulation of its counter-receptor triggering receptor expressed on myeloid cell-like transcript 2 at tumour sites.. Immunology.

[13] Yan RH, Zhang GB, Sun J, Fu FQ, Zhang XG (2010). Expression of mouse B7-H3-Fc fusion protein and characterization of its bioactivity.. Xi Bao Yu Fen Zi Mian Yi Xue Za Zhi.

[14] Nagashima O, Harada N, Usui Y, Yamazaki T, Yagita H (2008). B7-H3 contributes to the development of pathogenic Th2 cells in a murine model of asthma.. J Immunol.

[15] Lupu CM, Eisenbach C, Kuefner MA, Schmidt J, Lupu AD (2006). An orthotopic colon cancer model for studying the B7-H3 antitumor effect in vivo.. J Gastrointest Surg.

[16] Zhang G, Wang J, Kelly J, Gu G, Hou J (2010). B7-H3 augments the inflammatory response and is associated with human sepsis.. J Immunol.

[17] Sun M, Richards S, Prasad DV, Mai XM, Rudensky A (2002). Characterization of mouse and human B7-H3 genes.. J Immunol.

[18] Steinberger P, Majdic O, Derdak SV, Pfistershammer K, Kirchberger S (2004). Molecular characterization of human 4Ig-B7-H3, a member of the B7 family with four Ig-like domains.. J Immunol.

[19] Kakoulidou M, Wang X, Zhao X, Pirskanen R, Lefvert AK (2007). Soluble costimulatory factors sCD28, sCD80, sCD86 and sCD152 in relation to other markers of immune activation in patients with myasthenia gravis.. J Neuroimmunol.

[20] Zhang G, Hou J, Shi J, Yu G, Lu B (2008). Soluble CD276 (B7-H3) is released from monocytes, dendritic cells and activated T cells and is detectable in normal human serum.. Immunology.

[21] Zhang G, Xu Y, Lu X, Huang H, Zhou Y (2009). Diagnosis value of serum B7-H3 expression in non-small cell lung cancer.. Lung Cancer.

[22] Chen X, Zhang G, Li Y, Feng X, Wan F (2009). Circulating B7-H3(CD276) elevations in cerebrospinal fluid and plasma of children with bacterial meningitis.. J Mol Neurosci.

[23] Zhang G, Wang J, Kelly J, Gu G, Hou J B7-H3 augments the inflammatory response and is associated with human sepsis.. J Immunol.

[24] Logue EC, Bakkour S, Murphy MM, Nolla H, Sha WC (2006). ICOS-induced B7h shedding on B cells is inhibited by TLR7/8 and TLR9.. J Immunol.

[25] Magistrelli G, Jeannin P, Herbault N, Benoit De Coignac A, Gauchat JF (1999). A soluble form of CTLA-4 generated by alternative splicing is expressed by nonstimulated human T cells.. Eur J Immunol.

[26] Nielsen C, Ohm-Laursen L, Barington T, Husby S, Lillevang ST (2005). Alternative splice variants of the human PD-1 gene.. Cell Immunol.

[27] Lai SW, Cheung TC, Chan MC, Cheung KK, Peng SM (2000). Luminescent mononuclear and binuclear cyclometalated palladium(II) complexes of 6-phenyl-2,2 bipyridines: spectroscopic and structural comparisons with platinum(II) analogues.. Inorg Chem.

[28] Tran CN, Thacker SG, Louie DM, Oliver J, White PT (2008). Interactions of T cells with fibroblast-like synoviocytes: role of the B7 family costimulatory ligand B7-H3.. J Immunol.

[29] Nei M, Xu P, Glazko G. (2001). Estimation of divergence times from multiprotein sequences for a few mammalian species and several distantly related organisms.. Proc Natl Acad Sci U S A.

[30] Zhou YH, Chen YJ, Ma ZY, Xu L, Wang Q (2007). 4IgB7-H3 is the major isoform expressed on immunocytes as well as malignant cells.. Tissue Antigens.

[31] Haack M, Enck S, Seger H, Geyer A, Beck-Sickinger AG (2008). Pyridone dipeptide backbone scan to elucidate structural properties of a flexible peptide segment.. J Am Chem Soc.

[32] Combet C, Jambon M, Deleage G, Geourjon C (2002). Geno3D: automatic comparative molecular modelling of protein.. Bioinformatics.

[33] Zhang GB, Chen YJ, Jiang Z (2004). Construction of humanB7-H3 transfected cell line and the investigation of its bioactivity in vitro.. Mod Immunol.

